# Predictability of Cardiovascular Risk Scores for Carotid Atherosclerosis in Community-Dwelling Middle-Aged and Elderly Adults

**DOI:** 10.3390/jcm13092563

**Published:** 2024-04-26

**Authors:** Chao-Liang Chou, Chun-Chieh Liu, Tzu-Wei Wu, Chun-Fang Cheng, Shu-Xin Lu, Yih-Jer Wu, Li-Yu Wang

**Affiliations:** 1 MacKay Medical College, New Taipei City 252, Taiwan; chaoliangchou@gmail.com (C.-L.C.);; 2 MacKay Memorial Hospital, Taipei City 104, Taiwan; 3 New Taipei City Government, New Taipei City 251, Taiwan

**Keywords:** carotid atherosclerosis, carotid ultrasonography, community-based study, Framingham Risk Score, Pooled Cohort Equations, risk prediction

## Abstract

**Background:** The assessment of future risk of cardiovascular diseases (CVD) is strongly recommended for all asymptomatic adults without CVD history. Carotid atherosclerosis (CA) is a preclinical phenotype of CVDs. However, data on estimated future CVD risks with respect to preclinical atherosclerosis are limited. This community-based study aimed to assess the relationships between predicted CVD risks and CA. **Methods:** We enrolled 3908 subjects aged 40–74 years without CVD history and calculated their 10-year CVD risks using the Framingham Risk Score (FRS) and the Pooled Cohort Equations (PCE). Carotid plaque (CP) at the extracranial carotid arteries was determined by high-resolution B-mode ultrasonography and further classified into mild or advanced CA. **Results:** The means of FRS for CP-negative and mild and advanced CA were 9.0%, 14.4%, and 22.1%, respectively (*p*-value < 0.0001). The corresponding values for PCE score were 4.8%, 8.8%, and 15.0%, respectively (*p*-value < 0.0001). The odds ratios (ORs) of having CP per 5.0% increase in FRS and PCE score were 1.23 (95% CI, 1.19–1.28) and 1.36 (95% CI, 1.28–1.44), respectively. The corresponding values of having advanced CA were 1.24 (95% CI, 1.19–1.29) and 1.38 (95% CI, 1.30–1.48), respectively. Among the models of FRS or PCE plus other conventional CVD risk factors, the FRS + age model had the highest discrimination for the presence of CP (AUROC, 0.7533; 95% CI, 0.7375–0.7691) as well as for the presence of advanced CA (AUROC, 0.8034; 95% CI, 0.7835–0.8232). The calibration of the FRS + age models for the presences of CP and advanced CA was excellent (χ^2^ = 8.45 [*p* = 0.49] and 10.49 [*p* = 0.31], respectively). **Conclusions:** Estimated future CVD risks were significantly correlated with risks of having CA. Both FRS and PCE had good discrimination for the presences of CP and advanced CA.

## 1. Introduction

Cardiovascular diseases (CVDs) are prevalent and widely recognized as one of the main health issues globally. The estimated number of global CVD cases rose from 271 million in 1990 to nearly double that in 2019 [1]. In 1990, CVDs were responsible for approximately 12 million deaths worldwide, which increased to 18.6 million in 2019 [1]. Furthermore, CVDs are significant contributors to disease burden, accounting for 15.5% (383 million) of global disability-adjusted life-years and 21.5% (307 million) of global years of life lost in 2019 [2]. The reduction in the impact of CVDs is critical and has significant implications for global health.

Etiological studies show that the male sex, older age, cigarette smoking, high blood pressure, dyslipidemia, hypertension, diabetes mellitus (DM), and obesity are correlated with significantly higher risks of CVD incidence and mortality [3,4,5,6]. Most of the previously mentioned major risk factors are closely related to lifestyle and are modifiable. The Global Burden of Disease Risk Factor study in 2013 showed that 88.5% of deaths, 76.5% of years lived with disability, and 87.9% of disability-adjusted life-years caused by CVDs were attributable to modifiable factors [7]. To promote primary and secondary CVD preventions, it is critical to calculate future CVD risks and formulate preventive programs according to the risk stratification. Consequently, many researchers of cardiovascular medicines have developed risk prediction models and algorithms for future CVD risks, such as the Framingham Risk Score (FRS), Systematic Coronary Risk Evaluation (SCORE), the Reynolds risk score, and the most recently Pooled Cohort Equations (PCE) [8,9]. These CVD risk assessment tools have been validated by prospective studies and demonstrated good abilities to discriminate between subjects who will and will not develop CVDs [9]. Consequently, professional societies highly recommended the assessment of future CVD risks for all asymptomatic adults without history of atherosclerotic CVDs [10,11]. The FRS and PCE are amongst the most frequently used CVD risk assessment tools and have been validated in East Asian populations [12,13,14].

Carotid atherosclerosis (CA) is a preclinical phenotype of CVDs and can be reliably detected by ultrasonography [15,16]. Significantly higher CVD risks in CA-positive subjects have been consistently demonstrated by large prospective studies [17]. More importantly, several studies have shown that CA measurement by ultrasound can improve the ability to predict CVDs [18,19,20,21]. It is reasonable to assume that future CVD risks may correlate with the presence of CA. However, there were only a few studies, mostly with small sample sizes or been conducted in DM patients, focusing on the impact of future CVD risks on the presence of CA. We therefore conducted this community-based study to evaluate the effects of future CVD risks on the presence and severity of CA.

## 2. Methods

### 2.1. Study Subjects

The study subjects were recruited from two community-based cohort studies that enrolled adults aged 40 to 74 years who resided in the northern coastal area of Taiwan [22]. Cohort I and II enrolled study subjects from September 2010 to May 2011 and from September 2014 to May 2020, respectively. The enrollment of subjects was conducted at four sites, including one at Tamsui health center and three at schools, by batches. The duration of each batch was 4–5 months. A total of 4140 eligible residents provided informed consent voluntarily and completed questionnaire interviews, received physical examinations, and had their blood drawn for biochemical determinations at the date they provided informed consent. Subjects who completed physical and clinical examination but failed to complete carotid ultrasonography were excluded (*n* = 38). Additionally, subjects with a prior diagnosis of coronary artery disease (*n* = 165) or with poor quality ultrasound images (*n* = 29) were excluded, resulting in a final sample of 3908 subjects (1375 males and 2533 females) in the present study. 

Due to the limitation of the capacity of ultrasound scans, not all enrollees received ultrasound scans at the same date of physical and biochemical examinations. The mean (standard deviation [SD], maximum) interval between the dates of physical and biochemical examinations and ultrasound scans was 29.4 (24.5, 113) days. Approximately 90% of the intervals were less than 60 days and less than 1.0% of the intervals were greater than 90 days. 

### 2.2. Measurements of Anthropometric and Clinical Characteristics 

The measurements of baseline anthropometric characteristics, including body weight, body length, waist circumference, and hip circumference have been described previously [23,24]. In brief, we used a digital system (BW-2200; NAGATA Scale Co. Ltd., Tainan, Taiwan) to measure subject’s body weight and height. Waist circumference (WC) was measured at the level of mid-distance between the bottom of the rib cage and the top of the iliac crest. Hip circumference was the distance around the largest part of individuals’ hips. Body mass index (BMI) was calculated as (body weight in Kg)/(body height in meter)^2^ and used as an index of general obesity. Waist-to-hip ratio (WHR) was calculated as (waist circumference) × 100%/(hip circumference) and used as an index of central obesity. 

The measurements of baseline clinical characteristics, including systolic blood pressure (SBP), diastolic blood pressure (DBP), and fasting levels of total cholesterol (CHO), high-density lipoprotein cholesterol (HDL-C), low-density lipoprotein cholesterol (LDL-C), glucose (Glu), and triglyceride (TG) were described previously [23,24]. In brief, blood pressure was measured three times with a minimum gap of 3 min using a digital system (UDEX-Twin; ELK Co., Daejon, Republic of Korea). The mean of these measurements was used to determine SBP and DBP. A fasting blood sample was collected from each participant to determine their blood lipid and glucose levels. The levels of CHO, HDL-C, LDL-C, and TG were determined using an autoanalyzer (Toshiba TBA c16000; Toshiba Medical System, Holliston, MA, USA) and commercial kits (Denka Seiken, Tokyo, Japan).

In this study, cigarette smoking was defined as having smoked cigarettes at least 4 days per week during the past month before enrollment. Hypertension was defined as SBP ≥ 140 mmHg, DBP ≥ 90 mmHg, or a history of taking antihypertensive medications. Diabetes mellitus (DM) was defined as a fasting blood glucose ≥ 126 mg/dL or the use of insulin or other hypoglycemic agents.

### 2.3. Determination of Carotid Atherosclerosis (CA)

The method for measuring CA had been described previously [22,24]. Briefly, two experienced technicians operated high-resolution B-mode ultrasonography systems (GE Healthcare Vivid 7, Vivid E9, and Logie E; General Electric Company, Milwaukee, WI, USA) to obtain the transverse and cross-sectional images of extracranial carotid arteries according to the protocol recommended by the American Society of Echocardiography [25]. These ultrasonography systems were equipped with a multi-frequency linear array transducer. Images of the common carotid arteries (CCAs), bifurcation (BIF), internal carotid arteries (ICAs), and external carotid arteries (ECAs) were captured and digitally stored. To reduce measurement error, technicians who operated the ultrasonography systems and who measured the thickness of the carotid vessel walls were unaware of the clinical profiles of the examinees. In this study, a carotid plaque (CP) was defined as a focal protrusion 50% greater than the surrounding vessel wall, an intima-media thickness ≥ 1.5 mm, or local thickening ≥ 0.5 mm [26]. 

In this study, the number of CPs and the level of carotid stenosis in each carotid segment were evaluated. Carotid stenosis was calculated as the percentage of reduced diameter in the carotid arteries on either side, according to the criteria established by the European Carotid Surgery Trial [27]. In this study, advanced CA was defined as one large plaque (maximum diameter stenosis of 50% or more) or multiple plaques with at least one medium plaque (maximum diameter stenosis of 30 to 49%). Mild CA was defined as CP positivity but unfulfilled criteria of advanced CA.

### 2.4. Estimation of 10-Year CVD Risks

In this study, we used the “general CVD” algorithm proposed by D’Agostino et al. [28] to calculate the Framingham Risk Score (FRS). The “general CVD” algorithm is sex-specific and based on the findings of the Framingham Heart Study and the Framingham Offspring Study. The outcome events of the “general CVD” algorithms include coronary heart disease, stroke, peripheral artery disease, or heart failure. The PCE was proposed by the American College of Cardiology/American Heart Association in 2014 and based on the best available data from community-based cohorts [29]. The outcome events of the PCE include coronary heart disease death, nonfatal myocardial infraction, and fatal and non-fatal stroke. The participants of the Framingham Heart Study were basically Caucasian whites. There were sufficient participants of Caucasian whites and African–Americans in the PCE, yet there were insufficient numbers of other racial/ethnic groups. Therefore, for the PCE, we used the sex-specific equations for non-Hispanic whites to calculate a subject’s 10-year CVD risk. 

### 2.5. Statistical Analyses

In this study, we used the student’s *t*-test and one-way analysis of variance to examine the significance of difference in the means of continuous random variables among groups. Pearson’s chi-squared test was used to examine the associations between the distributions of categorical variables and the presence and severity of CA. All component factors for calculating FRS and PCE scores were included in multi-variable analyses to assess their relationships with CA. The strength of the relationships of the predicted future CVD risks with CA were assessed by logistic regression analysis and manifested by the odds ratio (OR) and its 95% confidence interval (95% CI). The abilities to discriminate between subjects with and without CP and between subjects with and without advanced CA were assessed by the area under the receiver operating characteristic curve (AUROC). The significance of the differences in the AUROC between tested and referent models were assessed by the Wald’s test. To assess the calibration of the prediction models, the predicted probabilities of having CA were categorized into deciles. The χ^2^ test was then used to evaluate the goodness of fit between the expected and the observed frequencies. All statistical analyses were performed using SAS 9.4 (SAS Institute Inc., Cary, NC, USA). 

## 3. Results

### 3.1. Baseline Clinical Characteristic and Prevalence of CP

In this study, all subjects had complete data on anthropometric and clinical profiles and results of carotid ultrasonography. The mean (SD) of age of the cohort members was 56.0 (8.9) years and 64.8% of them were females (Table 1). The means (SD) of FRS and PCE scores were 0.119 (0.115) and 0.070 (0.081), respectively. More than one-third of the study subjects had a positive image of an arterial plaque in their extracranial carotid arteries. The prevalence rates of mild and advanced CA were 21.7% and 12.7%, respectively. 

### 3.2. Comparisons between CP-Negative and CP-Positive Subjects

Table 2 shows that as compared to CP-negative subjects, CP-positive subjects had significantly higher means of age, BMI, WHR, blood glucose and pressure, and LDL-C and significantly higher proportions of male sex, cigarette smoking, hypertension, and DM. The mean of the HDL-C of CP-positive subjects was significantly lower than that of CP-negative subjects. The means (SD) of FRS and PCE scores of CP-positive subjects were significantly higher than those of CP-negative subjects (0.173 ± 0.138 vs. 0.090 ± 0.087 and 0.111 ± 0.097 vs. 0.048 ± 0.060; both *p* < 0.0001). 

Table 2 also shows that there were significantly linear trends in the severity of CA with respect to age, BMI, WHR, blood glucose and pressure, LDL-C, FRS, PCE score, male sex, cigarette smoking, hypertension, and DM. The trend in HDL-C with respect to the severity of CA was significantly negative. Subjects with advanced CA had significantly higher means (SD) of FRS and PCE scores than those with mild CA (0.221 ± 0.152 vs. 0.144 ± 0.121 and 0.150 ± 0.108 vs. 0.088 ± 0.082; both *p* < 0.0001). 

### 3.3. Association Analyses for the Presence of CP and Advanced CA

The results of multivariable analyses for the presence of CP are shown in Table 3. Model 1 was the best-fit model for the presence of CA. Older age, male sex, cigarette smoking, hypertension, DM, and elevated LDL-C were correlated with significantly higher likelihoods of having CP. The AUROC of the best-fit model was 0.7588 (95% CI, 0.7432–0.7744). Table 3 also shows that the age-adjusted ORs of having CP per 5.0% increase in FRS and PCE score were 1.23 (95% CI, 1.19–1.28) and 1.36 (95% CI, 1.28–1.44), respectively. As compared with the best-fit model, the AUROCs of FRS + age (Model 2) and PCE + age (Model 3) were slightly decreased.

Table 4 shows the results of the multivariable analyses for the presence of advanced CA. Similarly, the best-fit model contained age, sex, cigarette smoking, hypertension, DM, and LDL-C. The AUROC of the best-fit model was higher than those of the FRS + age model and PCE + age. The age-adjusted ORs of having advanced CA per 5.0% increase in FRS and PCE score were 1.24 (95% CI, 1.19–1.28) and 1.38 (95% CI, 1.30–1.48), respectively.

### 3.4. Calibration of the Prediction Models

**Figure 1:**
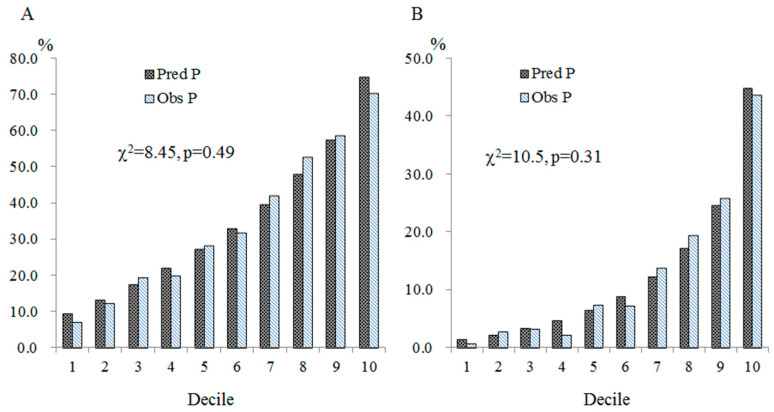
Calibration by decile for prediction of the presences of CP (**A**) and advanced CA (**B**). Vertical bars represent observed and model-based predicted prevalent rates. Note: Obs P, observed prevalence rate; Pred P, predicted prevalence rate.

The regression coefficients of the two FRS + age models were used to calculate the probabilities of having CP and advanced CA separately. To assess the calibration of the prediction models, the predicted probabilities were categorized into deciles. The average predicted probabilities for the presence of CP were 0.097, 0.135, 0.176, 0.223, 0.274, 0.331, 0.398, 0.480, 0.576, and 0.748 for the 1st to the 10th deciles, respectively. The observed frequencies of CP-positive subjects were 28, 49, 77, 78, 111, 125, 164, 206, 230, and 275, respectively. The calibration χ^2^ statistic was 8.45 (*p* = 0.49; Figure 1A).

The average predicted probabilities for the presence of advanced CA were 0.015, 0.023, 0.034, 0.048, 0.066, 0.090, 0.124, 0.173, 0.247, and 0.449 for the 1st to the 10th deciles, respectively. The corresponding observed frequencies of advanced CA were 3, 11, 13, 9, 29, 29, 54, 76, 101, and 171, respectively. The calibration χ^2^ statistic was 10.49 (*p* = 0.31; Figure 1B).

### 3.5. Discrimination Analyses for the Presence of CP

Table 5 shows the AUROC of the FRS- or PCE-score-only models and additions of other conventional CVD risk factors. Among the 24 models for the presence of CP, the AUROC was the highest for the FRS + age model (0.7533; 95% CI, 0.7375–0.7691), followed by the PCE + age model (0.7508; 95% CI, 0.7349–0.7666) and the PCE + SMK model (0.7476; 95% CI, 0.7316–0.7635), and was the lowest for the FRS + WHR model (0.7194; 95% CI, 0.7026–0.7362). As compared with the FRS-only model, the additions of sex, age, and SBP significantly improved the ability to discriminate between subjects with and without CP. The added AUROCs were 0.0045 (95% CI, 0.0005–0.0084), 0.0300 (95% CI, 0.0187–0.0414), and 0.0034 (95% CI, 0.0021–0.0047), respectively. As compared with the FRS + age model, further additions of other conventional CVD risk factors did not significantly increase the abilities to discriminate between subjects with and without CP (Supplementary Table S1).

As compared with the PCE-score-only model, the added AUROCs were non-significantly increased for the additions of sex, age, and SMK, were significantly decreased for the additions of SBP, LDL-C, and WHR, and were non-significantly decreased for other factors.

### 3.6. Discrimination Analyses for the Presence of Advanced CA

The AUROCs of the presence of advanced CA are also shown in Table 5. The AUROC was the highest for the FRS + age model (0.8034; 95% CI, 0.7835–0.8232), followed by the PCE + SBP model (0.8029; 95% CI, 0.0.7837–0.8221) and the PCE + sex model (0.8026; 95% CI, 0.7832–0.8220), and was the lowest for the FRS + WHR model (0.7704; 95% CI, 0.7491–0.7916). As compared with the FRS-only model, the discrimination capabilities for the additions of sex, age, and SBP were significantly improved. Further additions of other conventional CVD risk factors to the FRS + age model did not significantly improve the discrimination (Supplementary Table S2). 

As compared with the PCE-only model, the AUROC was significantly increased for the addition of SMK (added AUROC = 0.0013; 95% CI, 0.0001–0.0025). The additions of other conventional PCE CVD risk factors did not significantly influence the discrimination ability.

## 4. Discussion

In this study, we enrolled approximately 4000 middle-aged and elderly individuals and observed significant correlations between the estimated future CVD risks and CA. We found that both FRS and PCE score have good discrimination capacities for the presences of CA and advanced CA. Additionally, the discrimination and calibration of FRS and PCE scores improved when adding age to the prediction models. To our knowledge, only a few community- or population-based studies had explored the predictabilities of FRS and PCE scores on CA and no report had compared the discrimination capacities of FRS and PCE scores on the severity of CA before. 

In this study, the AUROC of the FRS-only and the PCE-only models for the discrimination between subjects with and without CA were 0.7233 and 0.7454, respectively. The corresponding values for the discrimination between subjects with and without advanced CA were 0.7736 and 0.8010, respectively. These values imply that both the FRS and PCE score have good capacities to discriminate between subjects with and without CP and between subjects with and without advanced CA. In this study, all subjects had no history of a prior CVD diagnosis and had never received carotid ultrasonography before. Therefore, it is reasonable to assume that the proportion of subjects with a positive CP image is a valid estimate of the lifelong cumulative incidence of CA. Accordingly, our findings can be interpreted as the FRS and PCE score being predictive of the lifelong cumulative incidence of CA. 

Additionally, as compared with the FRS-only model, the additions of several conventional CVD risk factors increased the discrimination capacities for the presence of CP and advanced CA. Similarly, as compared with the PCE-only model, the AUROC of the additions of sex, age, and SMK for the presence of CP and advanced CA were increased. As compared with the FRS + age model or the PCE + age model, the additions of other conventional CVD risk factors did not significantly improve the discrimination capabilities. Furthermore, we used the regression coefficients of the most predictive models, i.e., the FRS + age models, to calculate the probabilities of having CP and advanced CA for each subject. We categorized subjects into deciles, according to the estimated probabilities, and then assessed the degree of deviation between the observed and expected frequencies. The calibration χ^2^ statistics of the prediction models were excellent (Figure 1). This evidence indicates that to increase the discrimination and goodness of fit of the CVD risk assessment tools, it seems sufficient to add age to the employed tools. This evidence also indicates that, except for age, the effects of other FRS and PCE components on the incidences of CVD and CA are similar. Our speculations need further investigation.

In this study, we found that higher a FRS and PCE score were correlated with higher risks of having CP, and the ROC analyses showed that both the FRS and PCE score had good abilities to discriminate between subjects with and without CP. Our findings were supported by a few observational studies. A Korean study reported that among subjects affected with type II DM, CA was highly prevalent and linearly correlated with FRS [30]. Compared to subjects with an FRS Q1 score, the ORs of having CP for subjects with Q2, Q3, and Q4 scores were 1.21 (95% CI, 0.76–1.94), 2.18 (95% CI, 1.37–3.46), and 2.97 (95% CI, 1.86–4.74), respectively. Further analyses showed that the FRS had acceptable capability, with an AUROC of 0.633, to discriminate between subjects with and without CP [30]. A recent Argentine study reported a very good discrimination of FRS between the presence and absence of CP in DM patients without CVD history. The reported AUROC of the presence of CP was 0.822 (95% CI, 0.760–0.864) [31]. More recently, a cross-sectional study enrolled 120 adults aged 35–75 years and without history of any acute or chronic disease from a single center [32]. The presence rate of CP was 40% and the AUROC of the presence of CP was 0.799 (95% CI, 0.720–0.879) [32].

CVD is the outcome event of atherosclerosis; justifiably, our findings were supported by evidence from research on CVD. First, most CVD mortality and cases are attributable to traditionally biological and psychosocial factors [3,4,5,6]. For the primary prevention of CVD, a CVD risk assessment is of primary importance. The CVD risk assessment or prediction models are primarily based on the inclusion of multiple traditional and modifiable risk factors, e.g., blood pressure, blood levels of lipids and glucose, and anthropometric characteristics [33]. Among the proposed CVD risk prediction models, the FRS and the PCE are among the most frequently used algorithms and have been validated in East Asian populations [12,13]. A recent meta-analysis, which included 22 Chinese studies on CVD, reported that the overall C-statistics of FRS were 0.72 (95% CI, 0.71–0.84) and 0.74 (95% CI, 0.60–0.85) for males and females, respectively [14]. The corresponding values of PCE were 0.73 (95% CI, 0.69–0.77) and 0.76 (95% CI, 0.73–0.79), respectively. The authors concluded that the discrimination of FRS and PCE was acceptable and similar for males and females [14]. 

Second, carotid atherosclerosis is a preclinical phenotype of atherosclerotic cardiovascular diseases. Several prospective studies consistently demonstrated higher CVD risks in CA-positive subjects. The Rotterdam study, which enrolled 6389 subjects aged 55 or more and followed up for 7 to 10 years, demonstrates that four indicators of atherosclerosis predict myocardial infarction independently beyond traditional CVD risk factors. The multivariable-adjusted hazard ratio was 1.83 (95% CI, 1.27–2.62) for severe compared to no CA [34]. The Three City study enrolled 5895 elders who were free of coronary heart disease and identified 223 subjects with incident coronary heart disease after a median follow-up of 5.4 years [35]. As compared with CP-negative subjects, the multivariable-adjusted hazard ratios were 1.50 (95% CI, 1.0–2.2) and 2.2 (95% CI, 1.0–2.2) for subjects with CP at one and two sites, respectively [35]. In the Framingham Offspring study, the multivariable-adjusted hazard ratio was 1.92 (95% CI, 1.49–2.47) for CP-positive subjects compared to CP-negative subjects [18]. The BioImage study, which enrolled 5808 asymptomatic adults and identified 216 incident cases with major adverse cardiac events (MACE) after a median follow-up of 2.7 years, also showed significant trends between the multivariable-adjusted risk of MACE and carotid plaque burden [20]. A recent meta-analysis study, which included 2110 CP-positive and 10,237 CP-negative subjects from 11 prospective studies, showed a significant correlation between the presences of CP and silent brain infarction (OR = 2.78; 95% CI, 2.19–3.52) [36]. 

Finally, the additions of CA markers to traditional CVD risk factors improve the predictability of CVDs. In the Framingham Offspring study, the C-statistic significantly increased from 0.748 to 0.762 (*p* = 0.02) when adding CP at ICA to the regression model [18]. The net reclassification indexes were 1.8% and 5.8% in subjects without and with events, resulting in an overall net reclassification index of 7.6% (*p* = 0.01) [18]. Similar findings were demonstrated by several large prospective studies, including the IMPROVE study [21], the Multi-Ethnic study [19,37], the Three City study [35], and the ARIC study [38]. In addition, the addition of CA markers to traditional CVD risk factors also improves the predictability of CVDs in high-risk subjects. The APRES study, which enrolled 445 subjects without history or prior coronary artery but with a recent onset of chest pain, showed a significant difference in the prevalence rate of carotid plaques in subjects with and without obstructive CAD (71.5% vs. 34.8%, *p* < 0.001) [39]. The AUROC of FRS + CP for the presence of obstructive CAD was significantly larger compared with FRS only (0.7281 vs. 0.6689, *p* = 0.0019) [39]. More recently, a Chinese study, which enrolled 2149 subjects with a history of any type of chest pain, reported that the AUROC of the presence of CAD for a model with only traditional CVD risk factors (TRFs) was 0.761 (95% CI, 0.740–0.782) and significantly increased to 0.807 (95% CI, 0.785–0.826) for the TRF + CP model [40]. 

This study has several strengths and limitations. Previous studies that assessed the ability of CVD assessment tools to discriminate between subjects with and without CP mostly had small sample sizes and were conducted on DM patients. On the contrary, our study has a large sample size and enrolled subjects from communities who had never received a carotid ultrasound scan before and had no prior CVD history. This study has a statistical power greater than 0.99 to detect a mild association, e.g., a prevalence rate of 0.20 in the study group and an OR of 1.50. Therefore, our findings are more likely to reflect the natural spectrum. Additionally, in addition to the presence of CP, this study also explores the relationships between future CVD risk scores and the risks of advanced CA. However, the observational nature of the present study means that causal inference must be made with caution. In addition, due to the restriction of the Personal Information Protection Act, the district offices of household registration can only provide the address of the household with eligible subject(s). We are not able to know the detailed information, including age, sex, education, and other demographic characteristics, of eligible adults. As a result, we are unable to assess the representativeness and response rate of the study subjects. However, the primary aim of the study is to explore the predictive ability of estimated CVD risk scores for CA but not the prevalence rates of CA or other diseases. Furthermore, all subjects did not receive carotid ultrasound scan before enrollment and were unaware of the hypotheses of the study. Accordingly, our findings should be conservative.

## 5. Conclusions

We found that the estimated 10-year CVD risks significantly correlated with higher risks of having CP and advanced CA. Among the models for the presences of CP and advanced CA, the FRS + age model had the highest discrimination capabilities. As compared with the FRS + age model, the additions of other FRS components or other conventional CVD risk factors did not significantly improve the predictive abilities. Additionally, the calibrations of the FRS + age models for the predictions of having CP and advanced CA were excellent. Accordingly, it seems reasonable to use CA as an intermediate biomarker for preventive programs targeting atherosclerotic diseases. 

## Figures and Tables

**Table 1:** Baseline characteristics and segmental prevalence rates of carotid plaque in middle-aged adults and elders.

	All (*n* = 3908)	
	Mean	(SD)
Age at enrollment (years)	56.0	(8.9)
BMI (kg/m^2^)	24.6	(3.6)
WHR (%)	88.6	(7.1)
TC (mg/dL)	204.8	(38.6)
HDL-C (mg/dL)	55.6	(15.0)
LDL-C (mg/dL)	121.4	(32.5)
Triglyceride (mg/dL)	118.5	(89.8)
Glucose (mg/dL)	97.0	(25.2)
SBP (mm Hg)	126.5	(18.7)
DBP (mm Hg)	76.3	(12.6)
PCE (%)	7.0	(8.1)
FRS (%)	11.9	(11.5)
	n	(%)
Male sex	1375	(35.2)
Cigarette smoking	414	(10.6)
Hypertension	926	(23.7)
DM	420	(10.8)
Presence of carotid plaque	1344	(34.4)
Mild carotid atherosclerosis	847	(21.7)
Advanced carotid atherosclerosis	497	(12.7)

Notes: DBP, diastolic blood pressure; DM, diabetes mellitus; FRS, Framingham Risk Score; HDL-C, high-density lipoprotein cholesterol; LDL-C, low-density lipoprotein cholesterol; PCE, Pooled Cohort Equations; SBP, systolic blood pressure; SD, standard deviation.

**Table 2:** Comparisons of baseline clinical measurements between subjects with and without CP or among subjects with no, mild, and advanced CA.

	No CA		Presence of CA						*p*-Value of Significance Test	
	(A, *n* = 2564)		All (B, *n* = 1344)		Mild CA (C, *n* = 847)		Advanced CA (D, *n* = 497)			
Continuous variable	Mean	(SD)	Mean	(SD)	Mean	(SD)	Mean	(SD)	A vs. B ^1^	A, C, & D ^2^
Age (years)	53.6	(8.5)	60.7	(7.9)	59.1	(7.9)	63.4	(7.2)	<0.0001	<0.0001
BMI (kg/m^2^)	24.4	(3.6)	24.9	(3.4)	24.8	(3.5)	25.1	(3.3)	<0.0001	0.0017
WHR (%)	87.6	(7.1)	90.5	(6.8)	89.7	(7.1)	91.8	(6.1)	<0.0001	<0.0001
TC (mg/dL)	204.1	(37.6)	206.2	(40.3)	206.3	(38.5)	206.0	(43.2)	0.11	0.44
HDL-C (mg/dL)	56.6	(15.2)	53.9	(14.4)	55.0	(14.7)	52.0	(13.8)	<0.0001	<0.0001
LDL-C (mg/dL)	119.9	(31.7)	124.2	(33.9)	123.8	(32.5)	124.9	(36.3)	<0.0001	0.0086
TG (mg/dL)	116.9	(96.1)	121.7	(76.3)	118.9	(77.5)	126.5	(74.0)	0.11	0.074
Glucose (mg/dL)	95.0	(22.3)	100.9	(29.5)	98.1	(27.1)	105.5	(32.7)	<0.0001	<0.0001
SBP (mm Hg)	124.2	(18.4)	130.8	(18.4)	129.7	(18.4)	132.7	(18.3)	<0.0001	<0.0001
DBP (mm Hg)	75.7	(12.7)	77.6	(12.3)	77.3	(12.1)	78.2	(12.7)	<0.0001	0.0003
PCE (%)	4.8	(6.0)	11.1	(9.7)	8.8	(8.2)	15.0	(10.8)	<0.0001	<0.0001
FRS (%)	9.0	(8.7)	17.3	(13.8)	14.4	(12.1)	22.1	(15.2)	<0.0001	<0.0001
Categorical variable	n	(%)	n	(%)	n	(%)	n	(%)		
Male sex	774	(30.2)	601	(44.7)	337	(39.8)	264	(53.1)	<0.0001	<0.0001
Cigarette smoking	238	(9.3)	176	(13.1)	92	(10.9)	84	(16.9)	0.0002	<0.0001
Hypertension	429	(16.7)	497	(37.0)	268	(31.6)	229	(46.1)	<0.0001	<0.0001
DM	195	(7.6)	225	(16.7)	108	(12.8)	117	(23.5)	<0.0001	<0.0001

^1^, by using student’s *t*-test and Pearson’s chi-squared tests for continuous and categorical variables, respectively. ^2^, by using linear +regression analyses and the Mantel–Haenszel trend test for continuous and categorical variables, respectively. Note: BMI, body mass index; CA, carotid atherosclerosis; CP, carotid plaque; DBP, diastolic blood pressure; DM, diabetes mellitus; FRS, Framingham Risk Score; HDL-C, high-density lipoprotein cholesterol; LDL-C, low-density lipoprotein cholesterol; PCE, Pooled Cohort Equations; SBP, systolic blood pressure; TG, triglyceride; WHR, waist-to-hip ratio.

**Table 3:** Logistic regression analyses for the presence of CP.

	Model 1			Model 2			Model 3		
	Coeff. ^1^	OR	(95% CI)	Coeff. ^1^	OR	(95% CI)	Coeff. ^1^	OR	(95% CI)
Intercept	−7.3995	1.00		−5.5606	1.00		−4.8718	1.00	
Age (per 5.0 years)	0.4858	1.63 ***	(1.55–1.70)	0.3839	1.47 ***	(1.40–1.54)	0.3304	1.39 ***	(1.32–1.47)
Sex (M/F)	0.4596	1.58 ***	(1.35–1.86)	-			-		
Cigarette smoking (Y/N)	0.5380	1.71 ***	(1.33–2.20)	-			-		
Hypertension (Y/N)	0.5388	1.71 ***	(1.48–1.99)	-			-		
DM (Y/N)	0.5029	1.65 ***	(1.32–2.07)	-			-		
LDL-C (per 10.0 mg/dL)	0.0557	1.06 ***	(1.03–1.08)	-			-		
FRS (per 5.0%)	-			0.2094	1.23 ***	(1.19–1.28)	-		
PCE (per 5.0%)	-			-			0.3053	1.36 ***	(1.28–1.44)
AUROC (95% CI)	0.7588	(0.7432–0.7744)		0.7534	(0.7376–7692)		0.7509	(0.7350–0.7667)	

^1^, the regression coefficients of the logistic regression models. Note: AUROC, area under the receiver operating characteristic curve; CI, confidence interval; CP, carotid plaque; DM, diabetes mellitus; FRS, Framingham Risk Score; LDL-C, low-density lipoprotein cholesterol; OR, odds ratio; PCE, Pooled Cohort Equations; -, not included; ***, *p* < 0.0001.

**Table 4:** Logistic regression analyses for the presence of advanced CA.

	Model 1			Model 2			Model 3		
	Coeff. ^1^	OR	(95% CI)	Coeff. ^1^	OR	(95% CI)	Coeff. ^1^	OR	(95% CI)
Intercept	−10.9722	1.00		−8.4351	1.00		−7.2098	1.00	
Age (per 5.0 years)	0.6192	1.86 ***	(1.72–2.00)	0.4920	1.64 ***	(1.52–1.76)	0.3927	1.48 ***	(1.37–1.61)
Sex (M/F)	0.5913	1.81 ***	(1.45–2.26)	-			-		
Hypertension (Y/N)	0.6583	1.93 ***	(1.55–2.40)	-			-		
LDL-C (per 10.0 mg/dL)	0.0799	1.08 ***	(1.05–1.12)	-			-		
DM (Y/N)	0.7446	2.11 ***	(1.61–2.75)	-			-		
Cigarette smoking (Y/N)	0.8186	2.27 ***	(1.64–3.14)	-			-		
FRS (per 5.0%)	-			0.2142	1.24 ***	(1.19–1.29)	-		
PCE (per 5.0%)	-			-			0.3246	1.38 ***	(1.30–1.48)
AUROC (95% CI)	0.8152	(0.7964–0.8339)		0.8034	(0.7836–0.8232)		0.8010	(0.7811–0.8209)	

^1^, the regression coefficients of the logistic regression models. Note: AUROC, area under the receiver operating characteristic curve; CA, carotid atherosclerosis; CI, confidence interval; DM, diabetes mellitus; FRS, Framingham Risk Score; LDL-C, low-density lipoprotein cholesterol; OR, odds ratio. PCE, Pooled Cohort Equations; -, not included; ***, *p* < 0.0001.

**Table 5:** Discrimination analyses for the presences of CP and advanced CA.

	Presence of CP		Presence of Advanced CA	
	AUROC	(95% CI)	AUROC	(95% CI)
FRS	0.7233 ^1^	(0.7068~0.7398)	0.7736 ^4^	(0.7527~0.7945)
+Sex	0.7277 ^1,2^	(0.7113~0.7442)	0.7771 ^4,5^	(0.7564~0.7978)
+Age	0.7533 ^2^	(0.7375~0.7691)	0.8034 ^5^	(0.7835~0.8232)
+SMK	0.7269 ^1^	(0.7104~0.7434)	0.7766 ^4^	(0.7559~0.7974)
+DM	0.7234 ^1^	(0.7069~0.7399)	0.7745 ^4^	(0.7536~0.7954)
+HTN	0.7239 ^1^	(0.7074~0.7405)	0.7736 ^4^	(0.7528~0.7944)
+SBP	0.7267 ^1,2^	(0.7103~0.7431)	0.7840 ^4,5^	(0.7641~0.8039)
+CHO	0.7232 ^1^	(0.7066~0.7397)	0.7738 ^4^	(0.7529~0.7947)
+LDL-C	0.7209 ^2^	(0.7043~0.7375)	0.7724 ^4^	(0.7514~0.7934)
+HDL-C	0.7234 ^1^	(0.7068~0.7401)	0.7728 ^4^	(0.7514~0.7942)
+BMI	0.7259 ^1^	(0.7094~0.7424)	0.7758 ^4^	(0.7549~0.7966)
+WHR	0.7194 ^1^	(0.7026~0.7362)	0.7704 ^4^	(0.7491~0.7916)
PCE	0.7454 ^1^	(0.7294~0.7614)	0.8010	(0.7819~0.8202)
+Sex	0.7476	(0.7315~0.7636)	0.8026	(0.7832~0.8220)
+Age	0.7508 ^1^	(0.7349~0.7666)	0.8010	(0.7811~0.8208)
+SMK	0.7476	(0.7316~0.7635)	0.8023 ^6^	(0.7833~0.8214)
+DM	0.7453 ^1^	(0.7293~0.7613)	0.7998	(0.7805~0.8192)
+HTN	0.7443 ^1^	(0.7283~0.7603)	0.7992	(0.7799~0.8185)
+SBP	0.7435 ^1,3^	(0.7274~0.7596)	0.8029	(0.7837~0.8221)
+CHO	0.7443 ^1^	(0.7282~0.7603)	0.8010	(0.7819~0.8202)
+LDL-C	0.7406 ^1,3^	(0.7244~0.7568)	0.7987	(0.7792~0.8182)
+HDL-C	0.7453 ^1^	(0.7292~0.7614)	0.8007	(0.7813~0.8201)
+BMI	0.7460 ^1^	(0.7300~0.7620)	0.8004	(0.7809~0.8199)
+WHR	0.7380 ^1,3^	(0.7216~0.7543)	0.7953	(0.7754~0.8152)

^1^, significantly lower than that for the FRS + age model (AUROC = 0.7533). ^2^, significantly higher than that for the FRS-only model (AUROC = 0.7233). ^3^, significantly lower than that for the PCE-only model (AUROC = 0.7454). ^4^, significantly lower than that for the FRS + age model (AUROC = 0.8034). ^5^, significantly higher than that for the FRS-only model (AUROC = 0.7736). ^6^, significantly higher than that for the PCE-only model (AUROC = 0.8010). Note: AUROC, area under the receiver operating characteristic curve; BMI, body mass index; CA, carotid atherosclerosis; CHO, total cholesterol; CI, confidence interval; CP, carotid plaque; DBP, diastolic blood pressure; DM, diabetes mellitus; FRS, Framingham Risk Score; HTN, hypertension; HDL-C, high-density lipoprotein cholesterol; LDL-C, low-density lipoprotein cholesterol; PCE, Pooled Cohort Equations; SBP, systolic blood pressure; SMK, cigarette smoking; WHR, waist-to-hip ratio.

## Data Availability

The datasets used and/or analyzed during the current study are available from the corresponding author on reasonable request.

## Supplementary Materials

The following supporting information can be downloaded at: https://www.mdpi.com/article/10.3390/jcm13092563/s1, Table S1: ROC analyses for having carotid plaque; Table S2: ROC analyses for having advanced carotid plaque.
