# Supplementary material for: Predictability of Cardiovascular Risk Scores for Carotid Atherosclerosis in Community-Dwelling Middle-Aged and Elderly Adults
Source: J Clin Med. 2024 Apr 26;13(9):2563. doi: 10.3390/jcm13092563 (PMC11084830; doi:10.3390/jcm13092563)
Supplement: Supplementary file 1 [file jcm-13-02563-s001.zip › Supplementary Table S1.pdf]

Supplementary Table S1. ROC analyses for having CP.

Model	AUROC	(95% CI)	Δ AUROC	(95% CI)	p-value
FRS+Age	0.7533	(0.7375 ~ 0.7691)	Ref		
FRS+Age+Sex	0.7540	(0.7282 ~ 0.7697)	0.0007	(-0.0006 ~ -0.0019)	0.32
FRS+Age+Cigarette smoking	0.7532	(0.7375 ~ 0.7690)	-0.0001	(-0.0013 ~ 0.0011)	0.89
FRS+Age+DM	0.7533	(0.7375 ~ 0.7691)	<0.0001	(-0.0004 ~ 0.0005)	0.95
FRS+Age+Hypertension	0.7552	(0.7395 ~ 0.7709)	0.0019	(-0.0005 ~ 0.0044)	0.12
FRS+Age+SBP	0.7533	(0.7375 ~ 0.7691)	<0.0001	(-0.0006 ~ 0.0005)	1.00
FRS+Age+DBP	0.7535	(0.7377 ~ 0.7786)	0.0001	(-0.0004 ~ 0.0007)	0.60
FRS+Age+Total cholesterol	0.7534	(0.7376 ~ 0.7691)	0.0001	(-0.0005 ~ 0.0006)	0.87
FRS+Age+LDL-C	0.7541	(0.7383 ~ 0.7698)	0.0008	(-0.0011 ~ 0.0026)	0.41
FRS+Age+HDL-C	0.7533	(0.7376 ~ 0.7691)	<0.0001	(-0.0001 ~ 0.0001)	0.62
FRS+Age+BMI	0.7533	(0.7376 ~ 0.7691)	<0.0001	(-0.0002 ~ 0.0003)	0.79
FRS+Age+WHR	0.7539	(0.7382 ~ 0.7697)	0.0006	(-0.0009 ~ 0.0022)	0.42
