# Supplementary material for: Predictability of Cardiovascular Risk Scores for Carotid Atherosclerosis in Community-Dwelling Middle-Aged and Elderly Adults
Source: J Clin Med. 2024 Apr 26;13(9):2563. doi: 10.3390/jcm13092563 (PMC11084830; doi:10.3390/jcm13092563)
Supplement: Supplementary file 1 [file jcm-13-02563-s001.zip › Supplementary Table S2.pdf]

Supplementary Table S2. ROC analyses for having advanced CA.

Model	AUROC	(95% CI)	Δ AUROC	(95% CI)	p-value
FRS+Age	0.8034	(0.7835 ~ 0.8232)	Ref		
FRS+Age+Sex	0.8040	(0.7843 ~ 0.8236)	0.0006	(-0.012 ~ -0.0024)	0.52
FRS+Age+Cigarette smoking	0.8037	(0.7838 ~ 0.8235)	0.0003	(-0.0021 ~ 0.0026)	0.81
FRS+Age+DM	0.8043	(0.7845 ~ 0.8240)	0.0009	(-0.0009 ~ 0.0027)	0.33
FRS+Age+Hypertension	0.8049	(0.7852 ~ 0.8245)	0.0015	(-0.0011 ~ 0.0040)	0.25
FRS+Age+SBP	0.8035	(0.7837 ~ 0.8234)	0.0002	(-0.0009 ~ 0.0012)	0.73
FRS+Age+DBP	0.8035	(0.7836 ~ 0.8234)	0.0001	(-0.0009 ~ 0.0012)	0.80
FRS+Age+Total cholesterol	0.8039	(0.7842 ~ 0.8236)	0.0005	(-0.0006 ~ 0.0016)	0.36
FRS+Age+LDL-C	0.8051	(0.7856 ~ 0.8247)	0.0017	(-0.0007 ~ 0.0041)	0.15
FRS+Age+HDL-C	0.8034	(0.7837 ~ 0.8232)	0.0001	(-0.0010 ~ 0.0011)	0.90
FRS+Age+BMI	0.8033	(0.7834 ~ 0.8231)	-0.0001	(-0.0004 ~ 0.0002)	0.51
FRS+Age+WHR	0.8049	(0.7853 ~ 0.8245)	0.0015	(-0.0010 ~ 0.0041)	0.24
